# Recombinant ATPase of Virulent *Aeromonas hydrophila* Protects Channel Catfish Against Motile *Aeromonas* Septicemia

**DOI:** 10.3389/fimmu.2019.01641

**Published:** 2019-07-16

**Authors:** Hossam Abdelhamed, Michelle Banes, Attila Karsi, Mark L. Lawrence

**Affiliations:** Department of Basic Sciences, College of Veterinary Medicine, Mississippi State University, Starkville, MS, United States

**Keywords:** *Aeromonas hydrophila*, motile *Aeromonas* septicemia, aquaculture, recombinant vaccine, catfish

## Abstract

Channel catfish farming dominates the aquaculture industry in the United States. However, epidemic outbreaks of motile *Aeromonas* septicemia (MAS), caused by virulent *Aeromonas hydrophila* (vAh), have become a prominent problem in the catfish industry. Although vaccination is an effective preventive method, there is no vaccine available against MAS. Recombinant proteins could induce protective immunity. Thus, in this work, vAh ATPase protein was expressed, and its protective capability was evaluated in catfish. The purified recombinant ATPase protein was injected into catfish, followed by experimental infection with *A. hydrophila* strain ML09-119 after 21 days. Results showed catfish immunized with ATPase exhibited 89.16% relative percent survival after challenge with *A. hydrophila* strain ML09-119. Bacterial concentrations in liver, spleen, and anterior kidney were significantly lower in vaccinated fish compared with the non-vaccinated sham group at 48 h post-infection (*p* < 0.05). Catfish immunized with ATPase showed a significant (*p* < 0.05) higher antibody response compared to the non-vaccinated groups. Overall, ATPase recombinant protein has demonstrated potential to stimulate protective immunity in catfish against virulent *A. hydrophila* infection.

## Introduction

Aquaculture is an approximately $1.2 billion industry, and catfish production is a mainstay of the U.S. aquaculture industry, accounting for $386 million in 2016 (1). Disease outbreaks are among the primary limiting factors in catfish production (2). Infectious diseases account for the most significant percentage of losses, with around 65% of the fry and fingerlings lost during production (3). The three bacterial species responsible for most of these losses are *Edwardsiella ictaluri, Flavobacterium columnare*, and *Aeromonas hydrophila*. These pathogens are the causative agents of enteric septicemia of catfish (ESC), columnaris disease, and motile *Aeromonas* septicemia (MAS), respectively (4, 5).

Since 2009, a clonal group of *A. hydrophila* strains (referred to as virulent *A. hydrophila* or vAh) has become a major pathogen of farm-raised channel catfish, causing motile *Aeromonas* septicemia (MAS) outbreaks (6). The Aquatic Diagnostic Laboratory at Mississippi State University has reported a continued increase of vAh for the past 5 years. The disease is most common in summer months (7). Estimated losses in ponds with disease outbreaks of vAh infection ranged from 4,000 to 10,000 pounds lost (about 8,000–15,000 fish), and pond mortality rates can be very high (close to 100%). vAh is distinguishable from previous *Aeromonas* catfish isolates, but it is very similar to an Asian grass carp isolate (8). In the last decade, $60–70 million in losses to the U.S. aquaculture industry have been attributed to MAS outbreaks due to mortalities, lost feeding days, and costs associated with antimicrobial therapy (9).

The lack of preventive measures to control vAh infection has emphasized the need to develop techniques for disease prevention. Recombinant protein technology is a promising technology for development of vaccines against many human and animal pathogens (10, 11). To select potential vAh recombinant protein candidates for use as a vaccine, genomic sequences from vAh strain ML09-119 (CP005966.1) were assembled against the genome of *A. hydrophila* reference strain ATCC 7966^T^ (NC_008570), revealing that *A. hydrophila* ML09-119, along with all other sequenced vAh strains, contains specific unique outer membrane and secreted proteins (6). These proteins include pilin protein, fimbrial biogenesis outer membrane usher protein, TonB-dependent siderophore receptor protein, TonB-dependent transferrin receptor, OmpA-like protein, and ATPase. We postulate that these proteins could be effective in stimulating protective immunity in catfish against vAh infection.

vAh ATPase has 717 aa and contains two domains. The AAA (ATPases Associated with diverse cellular Activities) domain has 284 aa and is found in the AAA superfamily of ring-shaped P-loop NTPases, which exert their activity through energy-dependent remodeling or translocation of macromolecules (12, 13). The AAA superfamily of ATPases is found in all kingdoms of living organisms and catalyzes many cellular processes in which energy released from ATP hydrolysis is used in molecular remodeling functions (14). In bacteria, ATPases participate in diverse cellular processes including DNA replication, protein degradation, membrane fusion, microtubule severing, peroxisome biogenesis, signal transduction, and regulation of gene expression (15).

The second domain, putative AbiEii toxin domain, is a Type IV toxin-antitoxin (TA) system belonging to the nucleotidyltransferase superfamily (16). It is similar to proteins predicted to be members of the bacterial abortive infection (Abi) system, which enables bacteria to resist bacteriophage infection. Resistance strategies include promoting bacterial death, thus limiting phage replication within a bacterial population. There are 20 or more Abis, and they are predominantly plasmid-encoded lactococcal systems. The putative AbiEii domain is a type of TA system that functions by killing bacteria that lose the plasmid upon division. AbiE phage resistance systems function as novel Type IV TAs and are widespread in bacteria and archaea. Here, we describe the expression and purification of VAh ATPase protein (AHML_21010) and its immune stimulation properties to protect channel catfish against vAh infection.

## Materials and Methods

### Ethics Statement

Catfish experiments were performed according to guidelines of an approved protocol by the Institutional Animal Care and Use Committee at Mississippi State University.

### Bacterial Strains, Media, Plasmid, and Reagents

*Escherichia coli* strains NovaBlue (Novagen, Madison, WI, USA) and BL21 (DE3) (Invitrogen, Carlsbad, CA, USA) were used for cloning and expression, respectively. *E. coli* strains were cultured on Luria–Bertani (LB) agar or broth (Difco, Sparks, MD, USA) and incubated at 37°C throughout the study. *A. hydrophila* strain ML09-119 was cultured in brain heart infusion (BHI) agar or broth (Difco) and incubated at 30°C. Plasmid pET-28a (Novagen) was used as an expression vector. When required, isopropyl-β-D-thiogalactopyranoside (IPTG), kanamycin (Kan, 50 μg/ml), ampicillin (Ap, 100 μg/ml), and colistin (Col, 2.5 μg/ml) (Sigma–Aldrich, Saint Louis, MO, USA) were added to culture media.

### Cloning and Expression of ATPase Protein in *E. coli*

The coding region of ATPase (AHML_21010) was amplified from *A. hydrophila* strain ML09-119 genomic DNA by PCR using primers ATPaseF01 (AA**GGATCC**CAAGAGGGTGTTATGTCAGAGC) and ATPaseR01 (AA**GTCGAC**CCTGATGTCCAAGTTCATGTAT). Primers were designed using Primer3 (http://bioinfo.ut.ee/primer3-0.4.0/) based on the ML09-119 genome sequence and synthesized by Sigma-Aldrich. Amplified ATPase region was confirmed by sequencing. For cloning*, EcoR*I and *Sac*I restriction sites (bold letters) were incorporated into primers' 5′ ends. The amplified ATPase coding region (2,160 bp) was cloned into the *Eco*RI and *Sac*I restriction sites in pET-28a. Positive clones were selected on LB Kan plates and verified by colony PCR. ATPase sequence was confirmed using T3 and T7 terminator primers. *E. coli* BL21(DE3) competent bacteria were transformed by positive plasmid using chemical transformation and stored at −80°C in 20% glycerol.

For ATPase protein expression, LB broth containing Kan was inoculated with *E. coli* BL21 (DE3) (1:100) and cultured at 37°C with shaking at 200 rpm until OD_600_ reached 0.6–0.8, after which bacteria were induced with 1 mM IPTG and incubated for 8 more h. Whole bacteria proteins were solubilized in tricine sample buffer (Bio-Rad Laboratories, Hercules, CA, USA) for 5 min at 80°C, and protein separation was conducted using 12% SDS-PAGE to confirm expression of ATPase protein. Whole bacteria proteins from competent *E. coli* BL21 (DE3) and uninduced *E. coli* BL21 with recombinant clone were used as controls.

### Purification of Recombinant ATPase Protein

Recombinant ATPase protein containing six histidine tags (His6) was purified by His-Bind resin columns (Novagen). IPTG induced recombinant bacteria were harvested by centrifugation (12,000 x g for 20 min at 4°C), and pellets were lysed using Bug Buster protein extraction reagent (Novagen) with benzonase nuclease and protease inhibitor cocktail set III (Sigma). Soluble fractions were removed by centrifugation, and recombinant ATPase protein was purified from inclusion body pellets by suspending in lysis buffer (Tris-HCl buffer pH 8.0, 6 M urea) with gentle sonication (4 cycles, 10 s) on ice. After centrifugation, the recombinant protein was loaded onto a resin column (5 mL/column). The resin was washed with wash buffer (0.5 M NaCl, 60 mM imidazole, 20 mM Tris-HCl, pH 7.9), and eluted using elution buffer (1 M imidazole, 0.5 M NaCl, 20 mM Tris-HCl, pH 7.9). Elution fractions were collected for SDS-PAGE analysis. Quantification of the eluted ATPase fractions was determined on a spectrophotometer at 280 nm and Bradford assay (Bio-Rad) according to the supplier's instructions. The identity of recombinant ATPase protein was confirmed by MALDI-TOF mass spectrometry.

### Fish Vaccination

Specific pathogen free channel catfish (*n* = 300; mean weight: 68.77 g) were randomly stocked in 15 40-L tanks (20 fish/tank) supplied with flow-through dechlorinated municipal water and continuous aeration. Fish were acclimated for 1 week by feeding twice a day and monitoring water temperature (30°C) and water quality parameters. Fish were assigned to three groups randomly, and each group included five replicate tanks. Group A consisted of intraperitoneal injection of 100 μl of purified ATPase protein at concentration of 250 μg/ml emulsified with non-mineral oil adjuvant Montanide ISA 763 AVG (Seppic, Paris, France) at a ratio of 30:70 protein to adjuvant. Group B included fish injected with 100 μl of sterile phosphate buffered saline (PBS) emulsified with adjuvant, and group C included fish injected with 100 μl sterile PBS (sham-vaccinated). Fish were anesthetized with tricaine methanesulfonate (MS-222; Sigma) before handling.

At 3 weeks post-immunization, catfish were experimentally infected by bath immersion with 2.8 × 10^10^ CFU/ml of *A. hydrophila* ML09-119 for 6 h at 30°C (17). Bacterial infection dose was chosen based on previous experimental infection doses (8, 18). Bacteria numbers (CFU/ml) in the overnight cultures were determined by plating serial 10-fold dilutions on agar plates followed by viable colony counts. At 48 h post-infection, five fish from each group were euthanatized, and liver, spleen, and anterior kidney tissues were collected aseptically. Tissues were homogenized in 1 ml PBS, and tissue suspensions were diluted serially and spread in triplicate on BHI agar plates. Viable bacterial colonies were enumerated after incubating plates at 37°C for 48 h. The remaining ten fish in each group were monitored daily for 2 weeks to assess relative percent survival (RPS), which is calculated by [1– (% mortality of vaccinated fish / % mortality of control fish)] × 100 (19).

### Serum Antibody Response

Before and after immunization, blood was collected from the caudal vein of ten fish per group (two fish per tank), and after clotting the blood overnight at 4°C, serum was obtained by centrifugation at 3500 x g for 10 min.

Antibody titers were determined by enzyme-linked immunosorbent assay (ELISA) as described (20). In the whole-bacteria ELISA, 96-well Immulon™ plates (Bloomington, MN, U.S.A.) were coated with heat-killed whole bacteria (10^8^ CFU/ml) overnight at 4°C. For ELISA with purified protein, 96-well plates were coated with 100 μl/well of purified ATPase protein at a concentration of 20 μg/ml in PBS. Subsequently, wells were washed and blocked with 5% nonfat dry milk (Bio-Rad) in PBS for 1 h at room temperature. Wells were washed three times in PBS containing 0.05% Tween-20 (PBS-T). Diluted serum (1:100) was added to each well (50 μl /well), incubated for 1 h at 37°C, and washed with PBS-T. Fifty microliters of a 1:4 dilution of monoclonal antibody 9E1 (anti-catfish Ig) (21, 22) were added to each well. After 1 h incubation at 37°C, plates were washed with PBS-T, and goat anti-mouse antibody conjugate (Fisher Scientific, Pittsburg, PA, USA) was added. Plates were then incubated at room temperature for 1 h and washed. Finally, 100 μl of *p*-nitrophenyl phosphate substrate (Sigma 104 phosphatase substrate) dissolved in 10% diethanolamine buffer was added to each well, and plates were incubated for 45 min at room temperature. Absorbance at 405 nm was measured in an ELISA Microplate Reader (CA, USA). Control wells containing PBS buffer in place of serum were present in each plate and prepared in the same manner. To standardize, average background absorbance for each plate was subtracted from measured absorbance.

### Statistical Analysis

The effect of vaccination with ATPase protein on survival of catfish challenged with vAh was assessed with mixed model logistic regression using PROC GLIMMIX in SAS for Windows 9.4 (SAS Institute, Inc., Cary, NC, USA). The number of live catfish in a tank at the end of the trial was the outcome assessed using an events/trials syntax. Protein was the fixed effect assessed in the model. Tank within protein was included as a random effect in the model. The wild-type strain was the referent for comparisons of protein effect.

Effects of ATPase on the number of CFU in fish tissues and on antibody response were assessed by analysis of variance using PROC GLM. Separate models were used to assess CFU in liver, spleen, and anterior kidney as well as the ELISA results. The CFU data were transformed by first adding 1 to each CFU value and then taking the base 10 logarithm. ELISA data were transformed by taking the base 10 logarithm of each value. The distribution of the residuals was evaluated for each model to determine the appropriateness of the statistical model for the data. If the effect of protein was found to be statistically significant, least squares means were compared using the Dunnett adjustment for multiple comparisons with wild-type strain as the referent. A significance level of 0.05 was used for all analyses.

## Results

### ATPase Protein Purification

**Figure 1:**
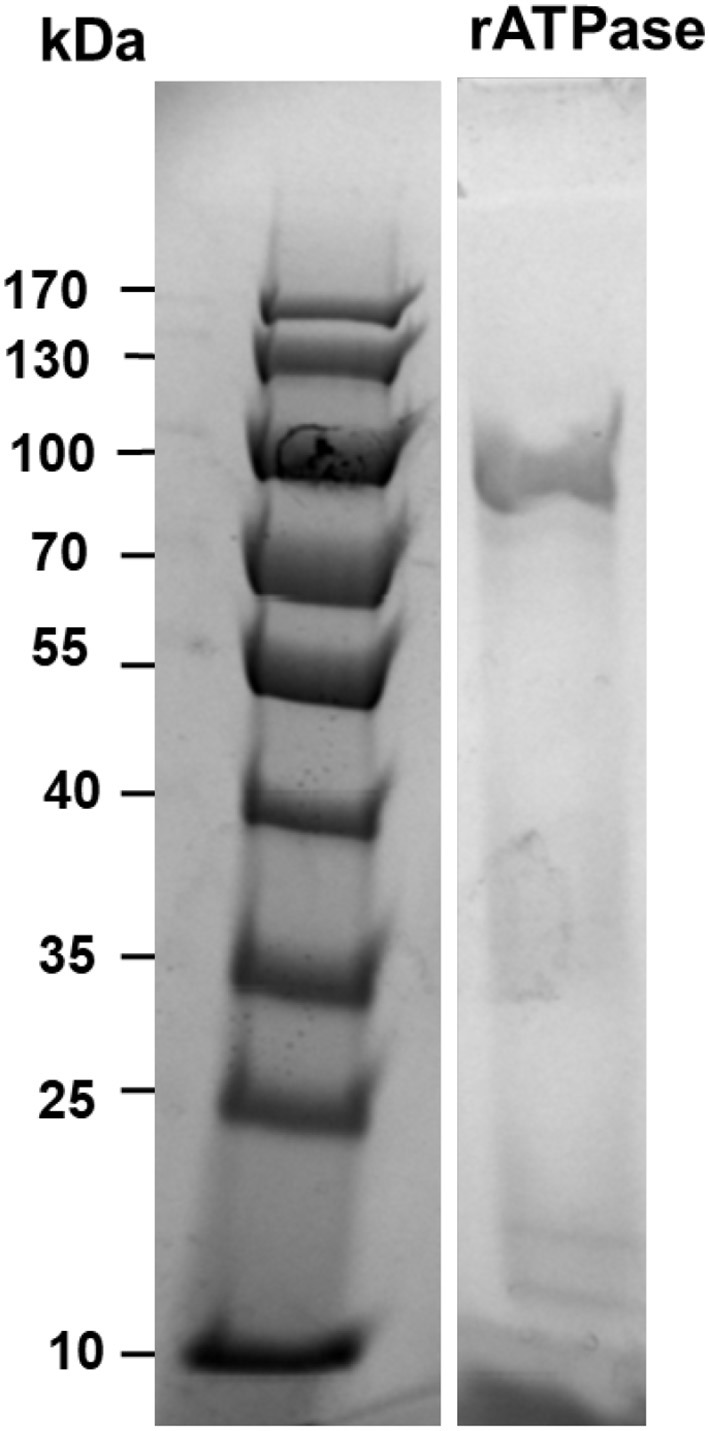
SDS-PAGE with Coomassie blue stain showing purified recombinant ATPase. Molecular weights in kilodaltons are shown for the standard protein marker in the right column. Intervening lanes between the molecular weight marker and the lane containing recombinant ATPase were removed.

The recombinant protein was purified successfully from soluble fraction at 0.2 mg/ml concentration, and amino acid sequences were confirmed by MALDI-TOF mass spectrometry. The SDS-PAGE result indicated the molecular mass of purified ATPase protein was approximately 81.5 kDa (Figure 1), which was the same size as the deduced molecular mass based on amino acid composition. Protein identification by peptide sequence using MALDI-TOF mass spectrometry revealed 97% identity of the purified protein to ATPase sequence (accession number: AGM45958).

### Fish Vaccination

**Figure 2:**
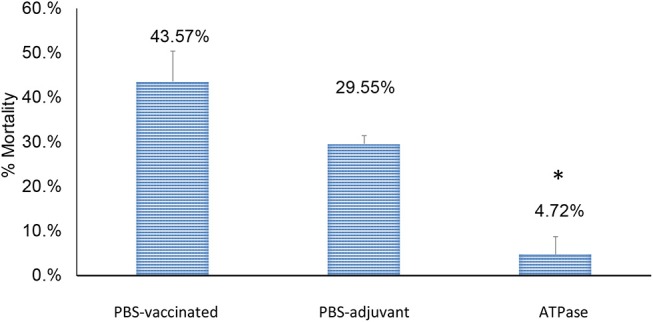
Percent mortalities in catfish vaccinated with recombinant ATPase protein following experimental infection with *A. hydrophila* ML09-119 at 3 weeks post-vaccination. Significant differences between treatments are indicated with asterisks (*p* < 0.05).

Catfish fingerlings immunized with recombinant ATPase protein showed 4.72% mortality (89.16% RPS), which was significantly lower (*p* < 0.01) than both non-vaccinated groups: PBS-adjuvant (29.55% mortality) and PBS-only (43.51% mortality) groups (Figure 2).

**Figure 3:**
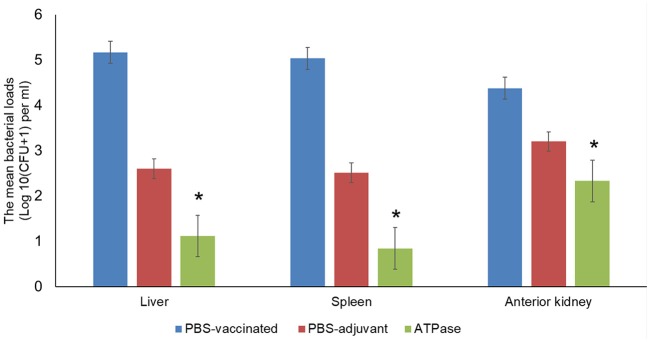
Mean bacterial concentrations (CFU/g) in liver, spleen, and anterior kidney of catfish vaccinated with recombinant ATPase protein at 48 h post-infection with *A. hydrophila* ML09-119. Data are presented as means ± SE (*N* = 5). Significant differences between treatments are indicated with asterisks (*p* < 0.05).

The mean number of viable bacteria in the liver, spleen, and anterior kidney was significantly lower in fish immunized with recombinant ATPase protein compared to non-vaccinated fish (*p* < 0.005) (Figure 3).

### Fish Serum Antibody Response

**Figure 4:**
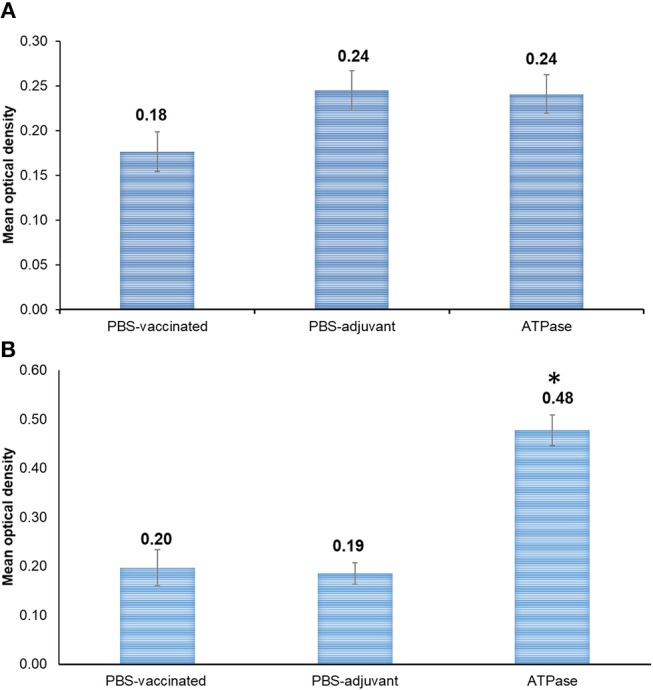
Antibody response in channel catfish serum at day 21 post-vaccination with recombinant ATPase protein. **(A)** Plates were coated with heat-killed whole bacteria. **(B)** Plates were coated with purified ATPase protein. Optical densities at 405 nm are means of 10 fish. Vertical bars denote standard errors of the mean. Significant differences are indicated with asterisks (*p* < 0.05).

There was no significant difference (*p* > 0.05) in antibody response between recombinant ATPase vaccinated and non-vaccinated catfish when ELISA plates were coated with whole bacteria lysate (Figure 4A). In ELISA plates coated with purified protein, significantly higher antibody titers were detected in serum of fish vaccinated with ATPase compared with PBS-only and PBS-adjuvant groups (Figure 4B).

## Discussion

This study aimed to determine the potential utility of recombinant ATPase protein as a possible vaccine against virulent *A. hydrophila*, an important pathogen responsible for MAS in catfish. Several research groups have identified candidate DNA or recombinant protein vaccines for MAS (23–28), but to date, there is no protective vaccine available against MAS caused by vAh. Previously, we purified four fimbrial proteins (FimA, Fim, MrfG, and FimOM) and three outer membrane proteins (major outer membrane protein OmpA1, TonB-dependent receptor, and transferrin-binding protein A) and assessed their ability to stimulate protective immunity in channel catfish fingerlings against vAh infection (17, 29). In the present study, expression and purification of vAh ATPase protein in *E. coli* were successful, and purified ATPase protein was recovered from the inclusion body.

*A. hydrophila* strain ML09-119 is representative of the vAh clonal group and exhibits high virulence in channel catfish (8, 30). Intraperitoneal injection of catfish with recombinant ATPase protein elicited a higher survival rate compared with non-immunized fish when challenged with vAh. Vaccination with recombinant ATPase protein also effectively reduced colonization of vAh in liver, spleen, and anterior kidney. No statistically significant difference was observed for antibody production in vaccinated vs. non-vaccinated fish when ELISA plates were coated with whole bacterial lysate. However, significant increase in antibody titer was detected in vaccinated fish when ELISA plates were coated with the purified recombinant protein, indicating that ATPase antigen concentration was insufficient in the whole bacterial lysate to detect ATPase-specific antibodies. In fish, antibody titers do not always correlate with protection (31). Protection generated by ATPase in our experiment could be mediated by antibody, and other factors could contribute such as cell-mediated immunity or innate immune components such as complement, lysozyme, antimicrobial peptides, or acute phase proteins (32–34). Innate immunity was stimulated in grass carp (*Ctenopharyngodon idella*) following immunization with F0F1 ATP synthase subunit beta. This was supported by a significant increase in the expression of pro-inflammatory cytokine genes in blood plasma, including IL-1β, IL-10, TNF-a, CRP, IFN, and MHC II (33). Innate immune response was significantly increased in rainbow trout (*Oncorhynchus mykiss*) infected with *Yersinia ruckeri* (35).

In some fish diseases, a combination of humoral, cell-mediated, and innate immune responses work in concert to provide protection. Recombinant outer membrane protein C of *Edwardsiella tarda* induced a significant innate immune response and humoral immune response in flounder (*Paralichthys olivaceus*); it also evoked significant protection against *E. tarda* challenge (36). Up-regulation of the immune-related genes encoding lysozyme G, complement factor 4, immunoglobulin M, β2-microglobulin, major histocompatibility complex I and II, and interleukin-1β was observed in Indian major carp *(Labeo rohita)* vaccinated with rOmpR, indicating that humoral, cellular, and innate immunity contribute to the protective response against *A. hydrophila* infection (34). In channel catfish, genes encoding iron homeostasis, transport proteins, complement components, acute phase response, and inflammatory and humoral immune response were upregulated following *E. ictaluri* infection, indicating a multifactorial catfish immune response against this pathogen (32).

Despite the considerable research on function of proteins in the AAA ATPases superfamily, there is not much information about their use as vaccine antigens or contribution to virulence (37). A few studies have utilized ATPase as a vaccine antigen against protozoan parasites in animal models. For example, DNA immunization of mice with Na^+^-K^+^ATPase from *Strongyloides stercoralis* induced protective immunity and a significant reduction in larval survival, thus suggesting that the Na^+^-K^+^ATPase may be a good potential target for the immune response (38). Vaccination with a recombinant chlamydial ATPase protein combined with Alum adjuvant resulted in reduction of the number of viable *Chlamydophila pneumoniae* in lungs of mice, indicating that chlamydial ATPase induces protective immunity in mice (39).

Another strategy investigated the use of ATPase for vaccine development in the form of fusion proteins. For example, a surface protein (TcSP2) of *Trypanosoma cruzi* fused to ATPase (ATP) domains of heat shock protein 70 (TcHSP70) induced high antibody titers and increased survival in immunized mice after *T. cruzi* infection (40). Moreover, TcHSP70, as well as an internal fragment of 242 amino acids within the ATPase domain, activated the maturation of dendritic cells macrophages to produce proinflammatory cytokines and chemokines in a mouse model (41).

No information is available on ATPase as a recombinant antigen in fish or other animals against bacterial pathogen infections. However, some proteins characterized by ATPase activity were successfully used as subunit vaccines against fish bacterial pathogens. For example, heat shock proteins (HSP 60 and HSP 70) have a N-terminal ATPase domain and are usually in an ATP bound state (42, 43). Wilhelm et al. (44) reported protection of salmon (95% RPS) against *Piscirickettsia salmonis* following immunization with HSP as a recombinant vaccine (44), and Sudheesh et al. (45) found that HSP60, HSP70, and two other proteins (ATP synthase and thermolysin) were highly immunogenic proteins against *Flavobacterium psychrophilum* (45).

In conclusion, ATPase was successfully expressed and purified using a pET-28a vector. The recombinant ATPase protein protected catfish against vAh infection and significantly reduced bacterial quantities in catfish tissue, and it stimulated significant antibody titers against the protein. This is the first study to report an ATPase protein as a potential vaccine for a bacterial disease in fish.

## Ethics Statement

Catfish experiments were performed according to guidelines of an approved protocol by the Institutional Animal Care and Use Committee at Mississippi State University.

## Author Contributions

ML, AK, and HA designed the experiments. ML supervised the overall project. HA performed the laboratory work, analyzed the data, and wrote the manuscript. MB helped with fish experiments and ELIZA. AK and ML reviewed the manuscript.

### Conflict of Interest Statement

The authors declare that the research was conducted in the absence of any commercial or financial relationships that could be construed as a potential conflict of interest.
